# Expanding the horizon of EV-RNAs: LncRNAs in EVs as biomarkers for disease pathways

**DOI:** 10.1016/j.vesic.2023.100025

**Published:** 2023-05-20

**Authors:** Michail Spanos, Priyanka Gokulnath, Emeli Chatterjee, Guoping Li, Dimitrios Varrias, Saumya Das

**Affiliations:** a Cardiovascular Research Center, Massachusetts General Hospital and Harvard Medical School, Boston, MA, USA; b Albert Einstein College of Medicine/Jacobi Medical Center, The Bronx, NY, USA

**Keywords:** Extracellular vesicles, Long-noncoding RNA, Cardiovascular disease, Cancer, Neurological disease

## Abstract

Extracellular vesicles (EVs) are membrane-bound nanoparticles with different types of cargo released by cells and postulated to mediate functions such as intercellular communications. Recent studies have shown that long non-coding RNAs (lncRNAs) or their fragments are present as cargo within EVs. LncRNAs are a heterogeneous group of RNA species with a length exceeding 200 nucleotides with diverse functions in cells based on their localization. While lncRNAs are known for their important functions in cellular regulation, their presence and role in EVs have only recently been explored. While certain studies have observed EV-lncRNAs to be tissue-and disease-specific, it remains to be determined whether or not this is a global observation. Nonetheless, these molecules have demonstrated promising potential to serve as new diagnostic and prognostic biomarkers. In this review, we critically evaluate the role of EV-derived lncRNAs in several prevalent diseases, including cancer, cardiovascular diseases, and neurodegenerative diseases, with a specific focus on their role as biomarkers.

## 1. Introduction

Fundamentally, all cells present in both eukaryotic and prokaryotic organisms communicate with each other through a plethora of intercellular communication strategies. Most cells harness this potential for rapid, effective, and precise biological cargo delivery by releasing nanosized, lipid bilayer membrane-enclosed packages called EVs into their surrounding environment. EVs can be retrieved from an array of biological fluids such as blood plasma, serum, urine, saliva, semen, breast milk, bile, ascitic, cerebrospinal, bronchoalveolar, and synovial fluid.^1–4^ EVs contain an intricate array of biomolecules not limited to but including protein, lipids, DNA, coding, and non-coding RNA (ncRNA). Owing to the phospholipid bilayer structure of EV membrane, fragile biomolecules, such as several RNA species that are otherwise prone to degradation, are sheltered from degradative enzymes, pH, and osmolar conditions that prevail in biofluids. In fact, EV contents can be transferred not only in a paracrine manner to the surrounding cells (horizontal transfer) but also to distant recipient tissues.^5,6^ Since EVs contain various populations, including certain functional RNA molecules, their role in genetically-encoded information exchange between cells has sparked in-depth scientific research in this direction and their impact at a cellular phenotypic level ought to be further explored.^7–9^ More importantly, the uptake of EV contents, specifically the transcriptomic cargo, could have profound implications for the recipient cells, as reflected by specific phenotypic alterations in their function or gene expression levels.^10–13^ Moreover, the cargo of vesicles is often subject to changes that are conferred upon different disease states and homeostatic perturbations and offer novel insights into the disease pathology. Although the EV cargo is composed of various molecules, non-coding RNAs have recently emerged as dynamic biomarkers.

**Fig. 1.**
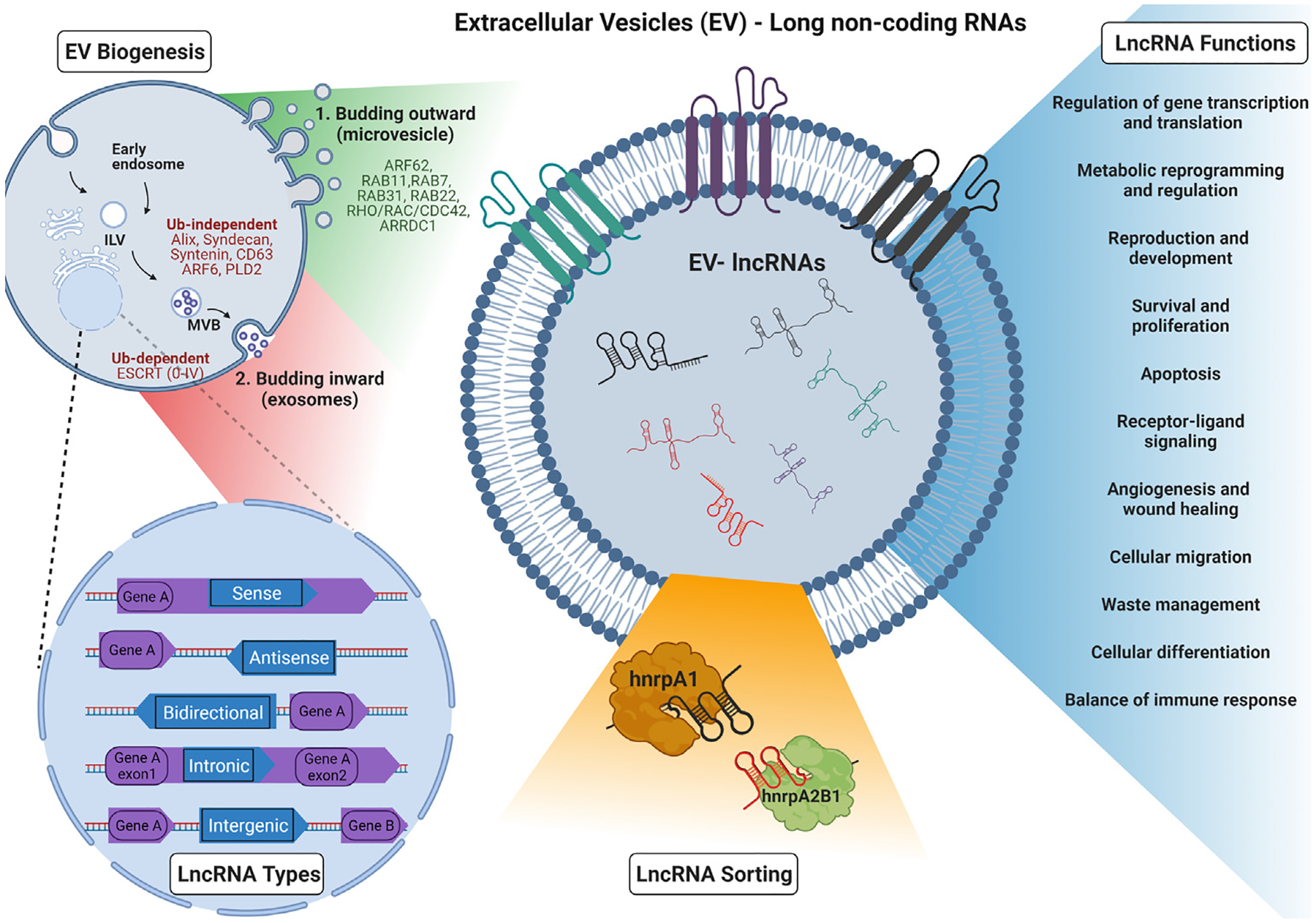
Extracellular vesicles-derived long non-coding RNAs — EV biogenesis, lncRNA types, sorting and function.

There is an entire repertoire of RNAs, called ncRNAs, defying the central dogma of biology, that are transcribed from the genome but do rarely encode for proteins. Though their discovery began as early as 1955 through the discovery of ribosomal RNA (rRNA) as a part of the Ribonucleoprotein (RNP) complex, the discovery of newer ncRNAs continues until today.^14^ The advent of technological advancements such as next-generation sequencing (NGS) paved the way for identifying an increasing number of these non-protein-coding sequences that account for about 98% of the human genome.^15,16^ Thus, a considerable part of our transcriptome is composed only of ncRNAs. NcRNAs are divided into (a) infrastructural ncRNAs such as transfer RNAs (tRNAs), ribosomal RNAs (rRNAs), small nuclear RNAs (snRNAs), small nucleolar RNAs (snoRNAs) and (b) regulatory ncRNAs including micro RNAs, circular RNAs (circRNA), enhancer RNAs, piwi RNAs, lncRNAs, Promoter associated RNAs, pseudogenes, the majority of which are comprised of lncRNAs.^17–19^ LncRNAs are a heterogeneous group of ncRNAs with more than 200 nucleotides, transcribed by RNA polymerase II (Pol II), capped/methyl-guanylated at the 5′ ends (m7G), many with polyadenylated at the 3′ ends and spliced like mRNA, but with longer and fewer exons.^20,21^ Next generation sequencing (NGS) has helped strand-specific, and allele-specific identification of lncRNAs, and newer such methodologies are consistently developed for more precise identification of lncRNAs.^22,23^ LncRNAs, though initially mistaken as pervasive transcripts, are crucial elements in cell regulation. Due to the lack of a better classification system, for ease of understanding, lncRNAs have been artificially categorized on the basis of origin from genomic location, mode of action, and function. Briefly, based on the position with respect to the genome (Fig. 1), there are (1) sense lncRNAs that overlap with the nearest protein-coding genes and are transcribed from the same strand; (2) antisense lncRNAs that are transcribed from the opposite strand of proximate protein-coding genes; (3) intronic lncRNAs, **t**ranscribed from the introns of protein-coding genes on both sense and anti-sense strands; (4) bidirectional lncRNAs that are transcribed in the opposite direction of the nearest protein-coding gene and lie generally within 1000bases (5) intergenic lncRNAs transcribed from regions between two protein-coding genes and account for most of the lncRNAs. LncRNAs have two modes of action and can regulate gene transcription via cis-acting (same region) or trans-acting (distant region) mechanisms based on where they are transcribed and where they act. LncRNAs are versatile molecules that have numerous functions ranging from reproduction, development, maintenance of cellular homeostasis with metabolic re-programming and regulation, cellular differentiation, migration, waste management and in specific processes such as receptor–ligand signaling, survival, apoptosis, angiogenesis, wound healing and balancing immune response as represented in Fig. 1.^24–29^ They are masters of genome regulation vital to both transcription and translation while contributing significantly towards epigenetic modulations. Given their role in genomic regulation, lncRNAs are abundantly found in the nucleus. However, they are also found in the cytoplasm, and their function usually depends upon their localization.^30^ For instance, in the nucleus, they act as decoys, scaffolds, guides, repressors, enhancers, enzyme modulators, Barr bodies, etc.,^31–33^ and in the cytoplasm, they play the role of competing endogenous RNAs, small RNA precursors, and RNAs bound to proteins that regulate translation.^32,34,35^ More recently, lncRNAs and their fragments have been identified in EVs with important roles in intercellular communication as discussed later.

In fact, lncRNA gene expression is usually cell-type specific, tissue-specific, species-specific and in many cases, disease-specific.^36–39^ Accumulating evidence has demonstrated that these molecules have a central role in the pathogenesis of many diseases; the presence of tissue- or disease-related lncRNAs or their fragments in EVs serve as an intriguing type of biomarker for the early detection of disease as a ‘liquid biopsy’. However, the biological role of circulating lncRNAs and the pathways regulating their levels still remain elusive. Nonetheless, many studies have reported that lncRNAs packaged in EVs, play crucial roles in various systemic human diseases, such as cancer,^40,41^ cardiovascular,^42,43^ infectious,^44,45^ and neurodegenerative diseases.^44,45^ Notably, EVs exhibit tissue specificity through their unique transcriptomic profile and can be traced back to the tissue-of-origin from a mixture of tissues/biofluids using computational algorithms on their transcriptional data.^46^ Moreover, another mode of identifying the tissue-specificity of EVs is through proteomic methods such as mass spectrometry.^47,48^ Although there is much to be gleaned in this field, it is interesting and relevant to understand the process of EVs biogenesis from cells and why certain transcripts are selectively packaged into EVs, directing them as coded messages for specified cellular functions. Many recent studies converge on the idea that small RNAs, such as micro RNAs (miRNAs), and tRNA-derived small RNAs^49^ are selectively packaged in EVs.^50^ However, the same cannot be said for long RNAs in EVs, which may be more of a stochastic process, hence can provide a useful ‘biopsy’ that can faithfully represent cellular transcriptome.^51^

## 2. Sorting and packaging of lncRNAs into EVs

International Society for Extracellular Vesicles (ISEV) defines EVs as particles naturally released from the cell that are delimited by a lipid bilayer and cannot replicate, i.e., lack a functional nucleus.^52^ EVs are sized from 40 nm to more than 1000 nm and can be variously categorized into different subtypes according to their biogenetic origin, size, physical characteristics, surface, or cargo marker expression and/or cell of origin. Current classification systems categorize EVs based upon their biogenesis, release pathways, content, and vesicular size. To name a few, based on biogenetic origin, EVs can be subcategorized into exosomes (vesicles derived from endosomes) and ectosomes (vesicles deriving from plasma membrane) whereas considering biophysical characteristics, three main types can be inferred: exosomes, microvesicles, and apoptotic bodies.^53–55^ It is certainly noteworthy that newer non-vesicular nanoparticles that may carry specific cargo are being discovered in certain contexts such as exomeres, and supermeres.^56–58^ These are recently described nanoparticles typically less than 50 nm in size and characterized by absence of lipid membrane and unique lipid and protein composition. Exomeres show enrichment for heat shock protein 90 alpha family class B member 1(HSP90AB1) while supermeres are enriched in several proteins that are associated with diseases such as transforming growth factor beta-1 (TGF*β*1-associated with colon cancer), amyloid precursor protein (APP-associated with Alzheimer’s disease), *α*-enolase and glypican-1 (associated with cancer), angiotensin-converting enzyme and angiotensin-converting enzyme 2 (ACE and ACE2 associated with cardiovascular disease and COVID-19).^59,60^

All currently described EV biogenesis pathways can be grouped into two main biosynthetic routes, both of which start with a membrane budding that encloses selectively sorted cargo in the lipid vesicle (Fig. 1). The first biogenesis pathway involves EVs that bud outward off the plasma membrane (e.g. ectosomes, sometimes referred to as microvesicles in literature),^61^ under the tight control of several proteins, such as ARF62, RAB11, RAB7, RAB31, RAB22, Rho/Rac/cdc42 and ARRDC1.^62,63^ The second biogenesis pathway starts with inward budding of endosomal membrane to form intralumenal vesicles (ILVs) within early endosomes that progressively mature into multivesicular bodies (MVBs) that, in turn, will fuse with the plasma membrane and be released (e.g. exosomes).^64^ For biomolecules to be sorted and transported into the ILVs, positive sorting signals are required. A positive sorting signal is a molecule which is added usually post-translationally and marks a specific cargo for a specific process, such as influx into an MVB in this instance. One such signal is the presence of a Ubiquitin (Ub) protein, and its addition is known as Ubiquitination.^65^ Recent research has shed light onto the sorting mechanisms that underlie the selective cargo incorporation into MVBs which can be further categorized as Ub-dependent and -independent. The Endosomal Sorting Complexes Required for Transport (ESCRTs) machinery is essential for Ub-dependent MVB biogenesis.^66^ The ESCRT system is comprised of a set of 4 interacting protein clusters, along with accessory proteins, that recognize ubiquitinated membrane proteins and promote their sorting into MVBs. ESCRT-0 (tumor susceptibility 101, TSG101) and ESCRT-I (Signal transducing adapter molecule 1) select cargo (TSG101 recognizes the Ub-tagged molecules)^67^ and trigger ESCRT-II/III recruitment and ILV formation, to complete vesicle formation after which a deubiquitinating enzyme removes the ubiquitin tag from the cargo proteins prior to final fission of the vesicle.^68,69^ Alix, Syndecan and Syntenin participate and interact within the ESCRT pathways prior to endosomal EVs release in Ub-independent MVB biogenesis. Syntenin binds syndecan to ALIX and aids in vesicle formation mediated by ESCRT-III.^70^ Selective cargo sorting of CD63 into vesicles and ALIX-Syntenin ILV formation pathway is regulated by the small GTPase ARF6 (ADP ribosylation factor 6) and the effector protein PLD2 (phospholipase D2).^71^ Another classification system which is based upon ESCRT involvement describes EVs as derived via an ESCRT-dependent or ESCRT-independent mechanism, however the pathways might be overlapping in some cases.^72^ Nonetheless, although in theory each EV sub-type stems from a distinct cellular compartment and exhibits distinct characteristics, recent evidence suggests there is considerable marker- and cargo-overlap between the different categories of EVs.^73^ Since defining a specific biogenetic pathway for an EV remains remarkably difficult; in this review, we use size to classify EVs and focus on small EVs (sEVs), that are <200 nm, while other EV categorizations are mentioned or discussed when relevant.

EVs carry an extensive repertoire of nucleic acids ranging from DNAs, mRNAs, and different types of ncRNAs such as rRNAs, tRNAs as well as their fragments, miRNAs, Y RNAs, snoRNA, snRNAs, piwi RNAs, circ RNAs, lncRNAs.^74^ It should be noted that there are other stable mechanisms of RNA transport in the plasma apart from EVs when bound to RNA binding proteins (RBPs) such as lipoproteins, and argonaute proteins.^74^ While most small RNAs are present in their full-length form in the EVs, long RNAs such as mRNAs and lncRNAs have been reported to be fragmented (although circ RNAs could remain intact in EVs).^75,76^ mRNA fragments have been reported to usually be 200 bases in length, with some extending even as long as 4 Kb.^77^ There have also been reports of lncRNAs loaded in EVs to have a mean size of 700 nucleotides.^78^ Considering the tissue-specific EV transcriptomics as mentioned earlier,^79^ one might infer that the loading of the lncRNAs or their fragments has a specific mechanism. Several studies probing into the mechanism of loading of miRNAs into EVs concluded that the overexpression of miRNAs in cells also correspondingly increases their levels in the EVs.^80^ While it is natural to expect lncRNAs that are abundant in cells to also be abundant in EVs, as observed in a previous study where there was a high correlation between EV-RNAs and cellular RNAs upon stress,^51^ there have been reports of the opposite being observed.^81^ Here, the authors report a selective abundance of certain lncRNAs in EVs even though they were less expressed in the parent cell line suggesting that these lncRNAs were distinctively loaded onto the EVs.^81^ Although this avenue is still being explored, there are a few studies that have provided an insight into this process.^82^ A heterogeneous ribonucleoprotein A2B1 (hnRNPA2B1), also as m6a reader, has been reported to be involved in the sorting of several lncRNAs, mostly in cancers, based on particular motifs such as GGAG-containing.^83–85^ In fact, an EV-lncRNA called lymph node metastasis-associated transcript 2 (LNMAT2) that has been reported to be involved in bladder cancer for promoting lymphangiogenesis, was selectively loaded onto EVs after binding with hnRNPA2B1.^86^ This RNA-RBP complex was also involved in the functionality of LNMAT2 when transported into recipient cells by locating to nucleus, further mediating H3 lysine 4 trimethylation to activate the PROX1 gene, thereby contributing towards metastatic phenotype. Similarly, in a study by Chen *et* al, the lncRNA EV-mediated LN-associated transcript 1 (ELNAT1) has been reported to be selectively loaded onto the EVs of bladder cancer cells through the ESCRT pathway when the RBP heterogeneous ribonucleoprotein A1 (hnRNPA1) residue underwent small ubiquitin-like modifier (SUMO) binding or SUMOylation at 113 lysine residue.^87^ It is pertinent to mention that the hnRNP family of proteins are important RBPs, primarily present in the nucleus, are involved in various cellular processes ranging from pre-mRNA or RNA processing to RNA splicing, RNA packaging, and RNA modifications. One or more members of this family are likely to be involved in the sorting of lncRNAs in EVs, and this warrants further investigation.^88,89^ Another study found that the RBPs YB-1 and NSUN2 could bind specifically to selected motifs, namely, ACCAGCCU, CAGUGAGC and UAAUCCCA in mRNAs and lncRNAs and selectively direct them to EVs.^90^ However, the selective lncRNA loading onto EVs warrants extensive research to precisely understand how and why certain lncRNAs are found in specific disease states and whether the RBP loading system holds true for most EV-lncRNA transport should be further explored.

Having summarized the biogenesis and sorting of EV-lncRNA, we briefly describe the most popularly accepted methods to study EVs and EV-derived lncRNAs. EVs are usually isolated using ultracentrifugation, differential ultracentrifugation, size exclusion chromatography, and commercially available EV-isolation kits. EVs, after isolation, are first examined for their sizes using particle analyzers such as nano particle tracking analysis (NTA), Spectradyne; the EVs could be visualized using Transmission Electron microscopy or quantitative single molecule localization microscopy (qSMLM),^91^ and further characterized qualitatively using western blot assay for the presence of tetraspanins such CD9, CD63, CD81 and cargo proteins such as Alix, syntenin. LncRNAs present in EVs are confirmed usually using RT-PCR or digital PCR. Interestingly, detection of biofluid EV-derived lncRNA has been well optimized by long RNA-sequencing techniques.^92^ Their presence can also be verified using Northern blot, Fluorescence in Situ Hybridization, Molecular beacons, and microarray techniques.

The omnipresence of EVs in human tissue and biological fluids, coupled with unique transcriptional cargo provided by lncRNAs along with their combinatorial distinct tissue-specificity render EV-derived lncRNAs as ideal biomarkers for human theranostics. Here, we explore the role of these EV-lncRNAs in the context of clinical biomarkers in some of the most lethal diseases wherein early indicators have a critical life-saving function.

## 3. Cancer

Cancer is an extremely heterogeneous disease with more than 200 types in the human body,^93,94^ with increasing prevalence and mortality.^95^ This accentuates the need for important prognostic and diagnostic biomarkers that aid in the better clinical management of this disease. In fact, nearly 90% of cancer mortality is attributed to the metastatic disease as opposed to the primary tumor itself,^96,97^ largely owing to tumor microenvironment-driven intercellular communication in its progression.^98^ Interestingly, cancer cells may use EVs as carriers of oncogenic protein for their spread and metastasis, demonstrating the significance of EVs in the tumor milieu.^99,100^ Specifically, PD-L1 in tumor-derived EVs contributes to immune evasion, which is a critical hallmark of cancer.^101^ Thus, inhibitors of EV release or fusion are considered a potential therapy for many cancers,^102,103^ although the specific role of any EV-lncRNAs in this process is undetermined. Further studies to investigate if inhibition of EV-mediated lncRNA transfer to recipient cells from malignant cells can impact cancer progression are needed. However, this is complicated by certain unique features of cancer EVs. Firstly, EVs secreted by tumor cells are much greater in number compared to normal cells.^104^ Secondly, tumor-derived EVs are comprised of highly diverse populations (e.g., “oncosomes” that are quite specific for cancers.^57^) Here, we will discuss EV-lncRNAs based on their potential to serve either as diagnostic or prognostic biomarkers in the most lethal cancer types (specifically in lung cancer, colorectal cancer, and breast cancer), prioritized by their functional role in the cellular context, such as tumor progression, immune evasion, and chemoresistance, specifically. However, it is important to mention that the functional roles have mostly been assigned due to the cellular role of these lncRNAs, and there have been very few studies that have characterized the EV-derived lncRNA role.

**Fig. 2.**
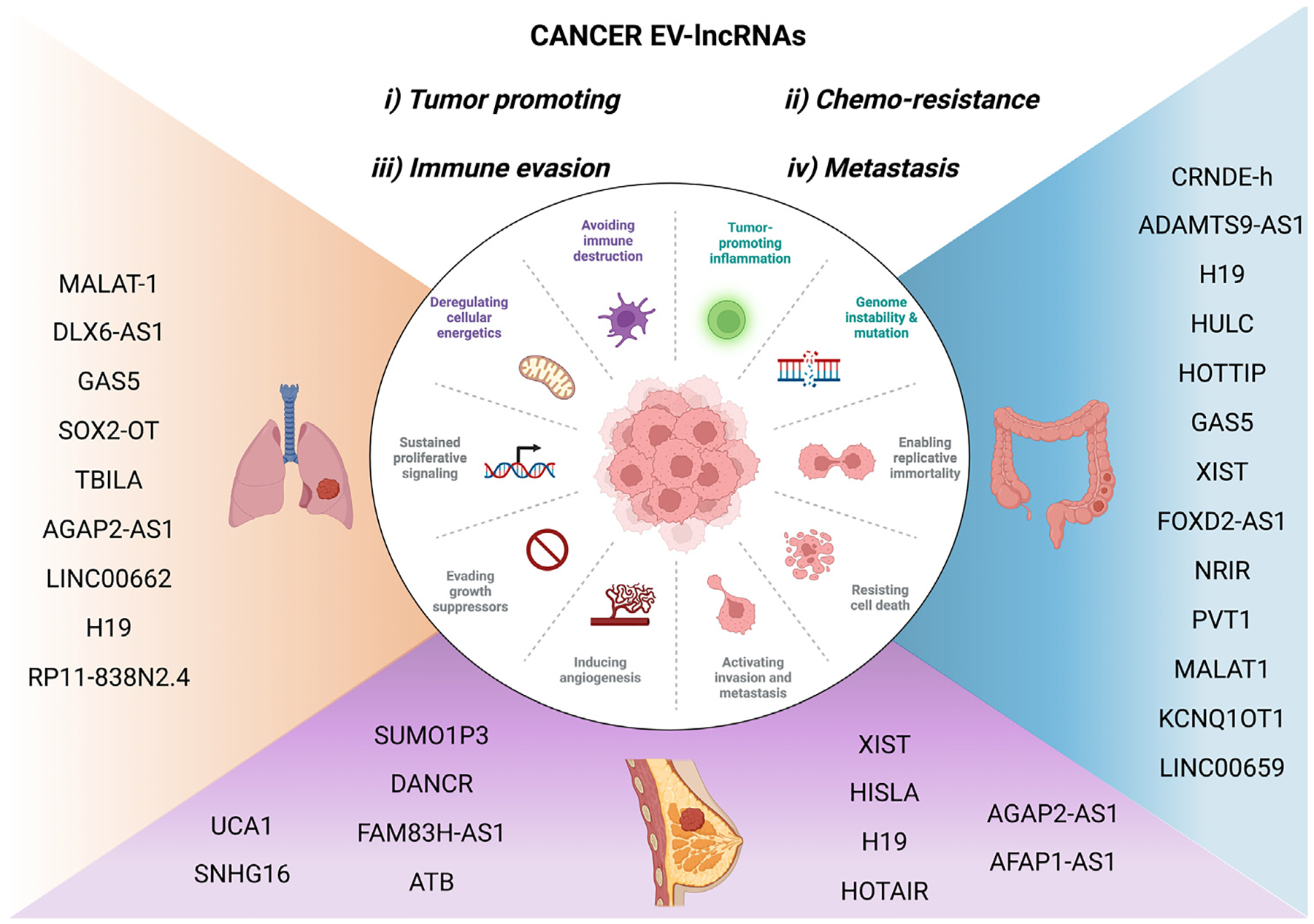
EV-lncRNAs in cancer.

Lung Cancer is one of the cancers with the highest mortality in men and women globally, with non-small cell lung cancer (NSCLC) accounting for most of it.^105^ NSCLC is usually diagnosed at later stages with an extremely poor prognosis,^106^ necessitating the identification of good early diagnostic markers with proper prognostic markers and therapeutic targets. Colorectal cancer (CRC) is the second most common cause of cancer-related mortality, after lung cancer, causing almost 935,000 deaths.^107^ Currently, the most commonly used biomarker is carcinoembryonic antigen (CEA) but with sub-optimal sensitivity and specificity. Therefore, given the very poor 5-year survival rate of CRC patients, better diagnostic markers that can detect disease at an early stage are still a necessity.^107–109^ Breast cancer (BC) is the most common cancer among women globally, accounting for 12% of all new cancer cases worldwide.^109–111^ While Cancer antigen 153 (CA153) and CEA are the commonly used biomarkers, there remains a need for better BC diagnostic and prognostic markers for BC. In each of these cancers, a few promising EV-lncRNAs biomarkers have been identified recently with important roles. However, we will try and limit the scope of discussion to those EV-lncRNAs (Fig. 2, Table 1) that have been validated in patient cohorts.

### (i) Markers of Tumor-progression

**Metastasis associated lung adenocarcinoma transcript 1 (MA LAT-1)**, a well-established lncRNA for its pivotal role as a promising prognostic marker in lung cancer,^142^ was also identified by Zhang et al. in 2017 to be expressed abundantly in serum EVs of NSCLC patients with an AUC of 0.703.^143^ In this study, serum EV-derived MALAT-1, analyzed using qPCR, was also positively associated with tumor stage and lymphatic metastasis. Further functional studies suggested that EV-associated MALAT-1 could stimulate tumor growth and migration while suppressing apoptosis *in vitro* in lung cancer cell lines as corroborated in another study.^144^ Further, its role in lung cells was examined *in vitro* and *in vivo* using knockdown and overexpression models to reveal that MALAT-1 acted as competing endogenous RNA to miR-515-5p, which in turn regulated the Eukaryotic Translation Elongation Factor 2 (EEF2) expression to aid in tumor progression. In another study, two serum EV lncRNAs **TGF-*β*** Induced **lncRNA** (**TBILA**) and **ArfGAP with GTPase Domain, Ankyrin Repeat And PH Domain 1 (AGAP2-AS1)** were detected to be increased in NSCLC patients compared to controls and individually had an AUC of 0.775 and 0.734 respectively.^145^ When these were combined with the commonly used serum tumor biomarker Cyfra21-1, the specific diagnostic accuracy for NSCLC patients enhanced considerably (AUC = 0.853). Interestingly, MALAT1 and AGAP2-AS1 were found to be elevated in EVs of smokers and NSCLC patients when compared to controls, indicating that these lncRNAs should be probed for their role as early diagnostic markers in larger cohorts. In BC, EV-derived MALAT1 was observed to be overexpressed in BC patients, correlated with poorer prognosis and clinical outcomes. This was brought about by possibly promoting cell proliferation, as observed by *in vitro* and *in vivo* experiments.^146^ Further, MALAT1 has been reported to be secreted by CRC tumor cells to mediate disease progression using their competing endogenous RNA (ceRNA) function by sequestering miR-26a/26b and regulating Fucosyltransferase 4 (FUT4) fucosylation to activate the PI3K/AKT pathway.^147^ While this study specifically proposes a role mediated by EV-derived MALAT-1, the validation on patient samples is indirect and only associative.

Serum EV-derived lncRNA **DLX6 antisense RNA 1 (DLX6-AS1)** was found to be elevated in 72 NSCLC patients compared to their 64 healthy controls and could be a promising diagnostic marker with AUC of 0.806.^148^ Not only was this lncRNA elevated in lung cancer patients in TCGA datasets, but more importantly, DLX6-AS1 levels in serum dynamically decreased post-operatively after tumor excision, suggesting that the enrichment of this lncRNA could be tumor-associated. In contrast, a serum EV-derived lncRNA **growth arrest specific 5** (**GAS5)** was observed to be downregulated in NSCLC patients with respect to control patients with an AUC of 0.857 and when it was combined with Carcinoembryonic Antigen (CEA, a conventional tumor marker) the AUC went up to 0.929 making this a very promising biomarker.^149^ Another study has implicated low EV-GAS5 to mechanistically promote angiogenesis in the tumor supported by *in vivo* analysis.^150^

### (ii) Markers of Chemoresistance

Given that drug resistance is a major factor that deters the effectiveness of cancer therapy, EV-lncRNAs such as **H19**^151^ and **RP11-838N2.4**^152^ have been observed to play a role specifically with erlotinib resistance having an AUC of 0.799 and 0.754 respectively as observed in NSCLC patient serum samples. EV-derived H19 has been recently implicated in gefitinib resistance of NSCLC as well.^153^ An EV lncRNA **HOXA Distal Transcript Antisense RNA (HOTTIP)** (oncogenic) was found to be a prognostic marker that can predict the relapse (AUC = 0.935, for 1st post-operative sample) by serially checking for this lncRNA in the serum of NSCLC patients from pre-surgical to post-surgical state (between 3–6 months).^154^

We now know that the tumor milieu is composed of cancer-associated fibroblasts (CAFs) and tumor-associated macrophages (TAMs) that form important components in aiding the cancer development, progression, metastasis, and drug resistance. This aspect was highlighted in a recent study wherein lncRNA **Long Intergenic Non-Protein Coding RNA 659** (**LINC00659)** was significantly upregulated in EVs derived from CAF rather than normal fibroblasts (NF) from CRC patients.^155^ This lncRNA was observed to be transferred to CRC cells, where it acted as a sponge for miR-342-3p to increase ANXA2 expression and promote cancer progression. In a similar mechanism, CAF-secreted colorectal cancer-associated lncRNA (CCAL) was observed to promote oxaliplatin (Oxa) resistance in CRC cells.^156^

Drug resistance and cancer recurrence are the principal sources of poor survival in breast cancer, and several EV-lncRNAs have been identified to play a role in promoting drug resistance. While circulating plasma-derived lncRNA **H19** was reported to be a promising biomarker (AUC = 0.81) of BC,^157^ serum EV-derived H19 was significantly elevated in BC patients compared to benign breast disease and control with an AUC of 0.87 as opposed to CEA153(0.822) and CEA (0.811).^158^ In this cohort, H19 also acted as a prognostic marker as it was associated significantly with Estrogen Receptor (ER), Progesterone Receptor (PR), human epidermal growth factor receptor-2 (Her-2), tumor node metastasis (TNM) stages, lymph node metastasis (LNM), and distant metastasis. Also, serum EV-derived H19 was observed to be elevated in non-responding patients (46) as opposed to responsive patients (36) after Doxorubicin (DOX) therapy with an AUC of 0.752 as evidenced by *in vitro* experiments as well.^159^ Another study,^160^ substantiated the predictive role of H19 after neo-adjuvant chemotherapy suggesting this lncRNA should be more carefully evaluated for drug resistance in larger cohorts. Serum EV-derived Small ubiquitin-like modifier 1 pseudogene 3 (**SUMO1P3**) was significantly elevated in Triple-Negative BC (TNBC), a subtype of BC.^161^ More importantly, this lncRNA also significantly decreased in chemosensitive patients after therapy, and high EV SUMO1P3 was associated with poor prognosis. Serum EV-associated lncRNA **AGAP2-AS1** is another trastuzumab resistance-predicting biomarker with an AUC of 0.784 and in cells, works through the ELAV like protein 1 (ELAVL1)/AGAP2-AS1/ATG10 axis to induce autophagy that in turn promotes drug resistance.^139^ Moreover, another lncRNA actin filament-associated protein 1-antisense RNA 1 (**AFAP1-AS1**) was identified in serum-derived EVs of trastuzumab non-responders.^162^ Han et al. demonstrated using *in vitro* and *in vivo* methods that trastuzumab-resistant tumor cells secrete AFAP1-AS1 taken up by the drug-sensitive cells where it binds to AU-binding factor 1 (AUF1) protein to promote the translation of er-BB2 receptor tyrosine kinase 2 (ERBB2) gene further conferring resistance in these cells. EV-derived **urothelial cancer associated 1** (**UCA1)** has been reported to confer tamoxifen resistance in BC cells *in vitro*.^163^ In 2018, Barbagallo et al. examined the expression of certain lncRNAs in CRC cell-line and biopsies along with EVs in cell-culture medium and serum samples of the same patients.^164^ Based on the extent of expression, their analysis yielded a set of lncRNAs differentially expressed at the cellular level and EV level. Interestingly, for several lncRNAs including **UCA1**, and Taurine Up-Regulated 1 (**TUG1** ) — were identified to have an inverse correlation with respect to expression, i.e., while UCA1 was upregulated in CRC tissues, it was downregulated in the EVs with respect to control. TUG1 followed the inverse pattern where it was more in EVs and downregulated in CRC tissues. Mechanistically, this suggests a selective retention or expulsion of the EV-lncRNA based on its tumor promoting or tumor suppressing role. However, the diagnostic efficacy estimated using the AUC curve for UCA1 was 0.719 while with TUG1, it was not significant. But on combining TUG1 and UCA1, their AUC went up to 0.814. In this study, UCA1 was found to aid in CRC progression by either binding to 3’ UTR of mRNAs (ANLN, BIRC5, IPO7, KIF2 A, and KIF23) to stabilize them or acting as ceRNA to miRNAs that degrade these mRNAs. Interestingly, a 2021 study^165^ corroborated the downregulation of circulating plasms UCA1 in 76 CRC patients with respect to 20 healthy controls. They confirmed that UCA1 levels helped identify BRAF mutation status in CRC patients. In another study,^166^ EV-derived UCA1 was found to be enriched in CRC patient tissues using microarray, and mechanistically in cells, it regulated the miR-143/MYO6 axis by acting as a ceRNA. While EV-UCA1 has been reported to be elevated in Controls compared to CRC patients, Yang et al. showed that EV-UCA1 levels increase upon cetuximab-resistant CRC as evidenced both *in vitro* and in CRC patients under cetuximab therapy.^167^

### (iii) Markers of Immune-evasion

Another tumor-promoting lncRNA **Ubiquitin-Fold Modifier- Conjugating Enzyme 1 (UFC1)**, was identified to be upregulated in both serum EVs and NSCLC tumor tissues of patients with an AUC of 0.812 in serum and 0.794 in serum EVs with a positive correlation with age and tumor infiltration.^168^ Using both *in vitro* and *in vivo* analysis, EV-transmitted UFC1 was noted to aid in cancer progression by binding to enhancer Enhancer of Zeste 2 Polycomb Repressive Complex 2 Subunit (EZH2) to promote epigenetic silencing of Phosphatase And Tensin Homolog (PTEN).^168^ **Colorectal neoplasia differentially expressed-h** (**CRNDE-h**) was a lncRNA that was identified in colorectal tissues. Its expression is both tissue-specific and age-specific where its expression is mainly during early development and minimally in adult tissues. In 2016, Liu et al.examined the serum EV-derived CRNDE-h expression in CRC patients (*n* = 148) and found that its expression was significantly upregulated when compared to controls (*n* = 80).^169^ Its biomarker potential (AUC = 0.892) was better than the traditional CEA biomarker (AUC = 0.688), with their combination being even better (AUC = 0.913). Given that T helper 17 cells (Th17) aid in tumor progression, Sun et al. showed that CRC tumor EV-derived CRNDE-h lncRNA is transmitted to CD4^+^ Tcells to increase the Th17 population and positively contribute toward CRC spread as seen *in vitro* and *in vivo*.^170^ In this study, CRNDE-h levels observed in the serum of CRC patients were noted to be positively correlated with Th17 cell proportion in tumor-infiltrating T cells.

EV-lncRNAs **KCNQ1 Opposite Strand/Antisense Transcript 1** (**KCNQ1OT1)** secreted by CRC tumor cells acted as a ceRNA on the miR-30a-5p/USP22 axis by regulating PDL-1 ubiquitination thereby inhibiting CD8^+^ T cell s.^171^ This autocrine effect mediates immune escape by CRC tumor cells and aids in CRC progression. One of the main limitations of this study is the indirect correlation demonstrated on the patient CRC tissues.

Tumor-infiltrating lymphocytes (TILs) have long been associated with the prognosis of BC, and depending upon which subtype forms the majority of TILs, their function could either be tumor-promoting or tumor-suppressing.^172^ Ni et al. identified that CD73^+^ V*δ*1 T cells were the most common regulatory T cells (Tregs) in BC (validated in 40 BC patient samples) and were also induced by them.^173^ BC tumor cells secreted EVs that contained **Small nucleolar RNA host gene 16** (**SNHG16**) that were taken up by V*δ*1 T cells in the tumor.^173^ In the EV recipient cells, SNHG16 acted as a miR-16-5p sponge that resulted in the increase of SMAD5 mRNA causing upregulation of the TGF beta pathway that further enhanced CD73 expression and activation of these Tregs.^173^ This process was hypothesized to promoted immunosuppression that in turn aided in cancer progression.

### (iv) Markers of metastasis

Plasma EV-derived **Growth Arrest Specific 5 (GAS5)** was analyzed between control (178) and CRC (153) patients, could distinguish between the two groups with an AUC of 0.964 and was observed to be associated with the TNM stage, LNM, local recurrence rate and distant metastasis rate. Interestingly, expression levels of its target miR-221 was also expressed in tissues, and plasma/EV level was associated with tumor size, TNM stage, LNM, local recurrence rate, and distant metastasis rate.^174^ Strikingly, plasma EV-derived lncRNA **SOX2 Overlapping Transcript (SOX2OT)** was specifically elevated in NSCLC patients with bone metastasis with increased expression correlating with a poorer prognosis. Mechanistically, **SOX2OT** acts as a ceRNA for miR-194-5p consequently increasing the expression of Rac Family Small GTPase 1 (RAC1) mRNAs as well as elevating the expression TGF-*β*, Parathyroid hormone-related protein (PTHrP) and receptor activator of nuclear factor-*κ*B ligand (RANKL) observed in bone lesions.^175^ Importantly, these studies show a pro-metastatic role for EV-derived SOX2OT on osteoclasts cells and mouse models. It is worth mentioning that plasma EV SOX2OT can also be used as a promising biomarker for Lung Squamous Cell Carcinoma with an AUC of 0.815.^176^

Apart from drug resistance, distal metastasis is a common cause of BC mortality, and the brain is one of the most common secondary sites for BC metastasis. EV-derived lncRNA **GS1-600G8.5** has been reported using *in vitro* and *in vivo* studies to be secreted by brain metastasis of BC cells, and it appears to compromise the integrity of the Blood-Brain Barrier further facilitating secondary tumors in the brain.^177^ Serum-derived FAM83H antisense RNA 1 (**FAM83H-AS1**) and lncRNA activated by TGF *β* (lncRNA **ATB**) were evaluated in 90 BC patients for their biomarker role. LncRNA ATB was found to be a more promising diagnostic given that the AUC values were 0.744 and 0.906.^178^ FAM83H-AS1 was observed to be a prognostic marker and showed a significant correlation with large tumor size, TNM stages, and LNM. Serum EV-associated **X-inactive specific transcript (XIST)** was found to be elevated in patients with TNBC (AUC = 0.888) and dynamically and significantly reduced after tumor resection.^179^ However, in patients showing recurrence, this lncRNA showed a marked increase and was also associated with a poorer prognosis. **HOX Transcript Antisense RNA (HOTAIR)**, identified by Tang et al. in serum-derived EVs was markedly elevated in BC patient samples compared to controls with an AUC of 0.916 as opposed to 0.7378 for CA153.^180^ Interestingly, HOTAIR levels decreased post-surgery suggesting that they vary dynamically in concordance with tumor mass; HOTAIR could also act as a prognostic marker as its high expression was associated with poor overall and disease-free survival and non-responsiveness to therapy. The biomarker role of HOTAIR in BC was confirmed by Wang *et* al in another study where it was noted to be correlated with ErbB2/HER2 positivity (*n* = 23).^181^ Another serum EV-lncRNA plasmacytoma variant translocation 1 (**PVT1**) was found to be elevated in colon cancer patients and the cellular PVT1 through miR-152-3p/VEGFA axis promoted stemness and metastasis of cancer.^182^

Although this was an attempt to categorize the EV-lncRNAs roughly based on their functional contribution toward certain lethal cancers, many of them have multiple roles. For example, EV-lncRNAs such as MALAT-1, and UCA-1 not only acted as early diagnostic markers but may also be involved in immune evasion, metastasis, and chemoresistance. Given their prominent role in cancer, it is certainly crucial to validate their detection and function in several patient cohorts. Another aspect worth pondering is that since many of these EV-lncRNAs are expressed in more than one type of cancer, could there be a common EV-lncRNA that could act as an early cancer detection biomarker. However, one must bear in mind that many of these are also expressed in other disease conditions, raising questions about their specificity for particular diseases. It is possible that these are secreted due to a particular cellular dysfunction that coincides with multiple disease conditions and, unfortunately, cannot solely play a role of a biomarker of a particular disease. An alternate would be to find a signature of EV-lncRNAs that comprise more than one candidate that can uniquely identify or mark a disease. In fact, if a discovery cohort spanning multiple cancer types carefully assess common EV-lncRNAs across different cancers, a panel of specific set of EV-lncRNA signature that can act as a pan-cancer early detection biomarker is a possibility. However, it is imperative that this is objectively assessed in multiple and large patient cohorts to qualify as a disease biomarker for clinical applications in the future.

## 4. Cardiovascular diseases

**Fig. 3.**
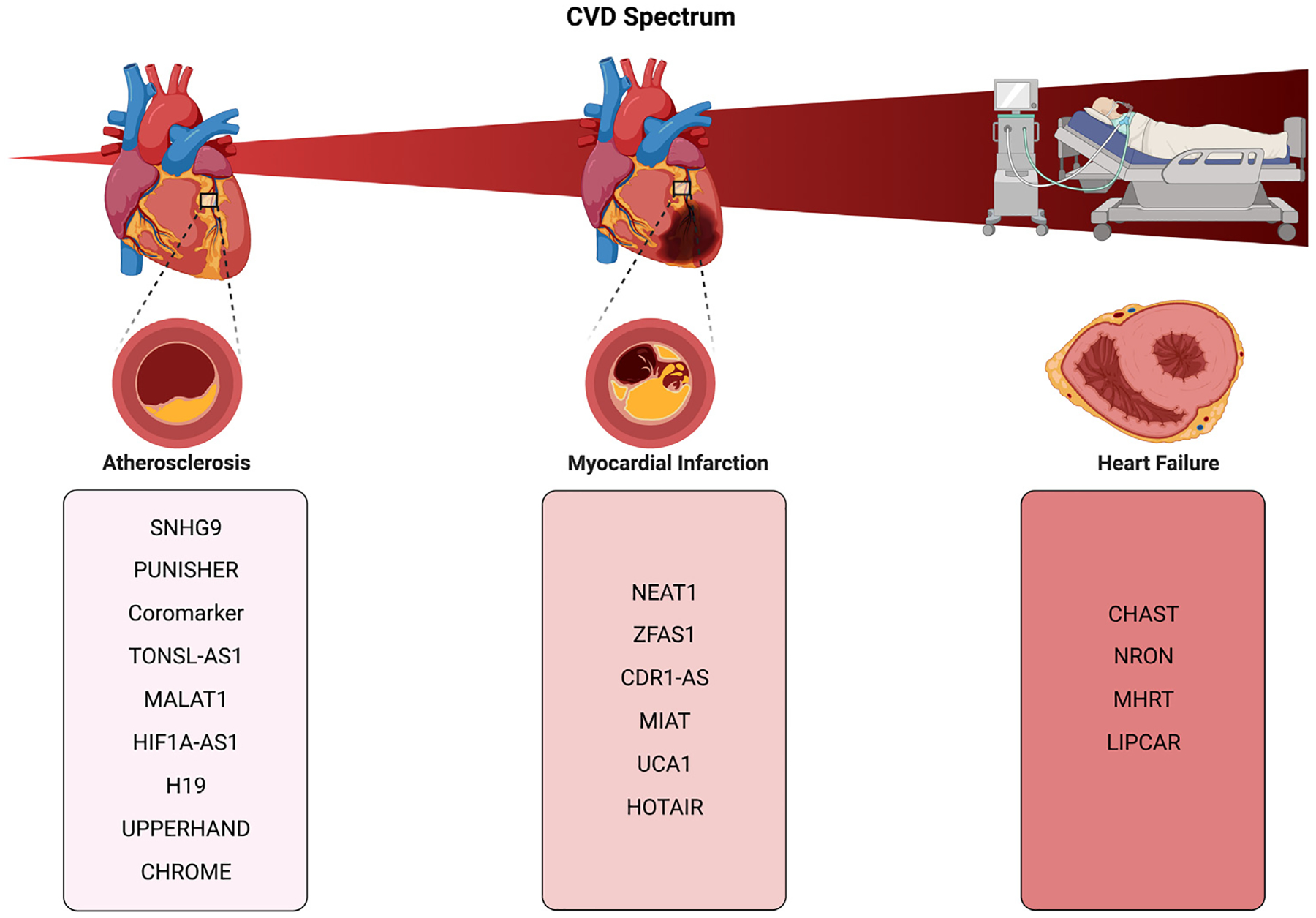
EV-lncRNAs in cardiovascular diseases.

Cardiovascular diseases (CVD) have an ever-increasing contribution to world-wide mortality and healthcare costs.^183–185^ Clinical biomarkers are an integral part of the diagnostic and prognostic cardiovascular workup for many CVDs, including atherosclerosis (AS), coronary artery disease (CAD), acute myocardial infarction (AMI), and heart failure (HF). However, even though several protein biomarkers for CVD (such as Creatine Kinase-MB, Natriuretic peptides, N-terminal fragment, and high-sensitivity cardiac troponin) have been incorporated into cardiovascular risk assessment scoring systems, specific and sensitive circulating biomarkers that can serve in CVD diagnosis, prognosis, and treatment response prediction are still the need of the hour. Recent studies in the cardiovascular field have shown that dysregulated lncRNAs are associated with different kinds of CVDs, as part of the underlying pathophysiologic pathways, revealing their potential as novel therapeutic targets, probes, and biomarkers of disease. Lately, extracellular lncRNAs which are incorporated in sEVs, have attracted vast research traction mainly due to their tissue-specificity and their detectability from all sorts of human biofluids.^186^ EV-lncRNAs (Fig. 3, Table 2) show great potential in enhancing or even replacing the traditionally used clinical biomarkers in the future.

### (i) Atherosclerotic cardiovascular disease (ASCVD)

Atherosclerotic CAD culminating in acute coronary syndrome or myocardial infarction is a central contributor to CVD morbidity and mortality, with about 20% of patients with myocardial infarction having fatal outcomes within the first month of the event.^231^ Endothelial injury and inflammation are considered the initial events in the development of AS and CAD. EV-lncRNAs have been shown to exert regulatory effects and thus actively participate in endothelium homeostasis.^232^ EV-lncRNA, **PUNISHER** (also known as AGAP2-AS1) was shown to act on the endothelial cells after being loaded on sEVs via the RNA-binding protein (RBP), heterogeneous nuclear ribonucleoprotein K (hnRNPK), and modulate the expression of the proangiogenic protein vascular endothelial growth factor A (VEGFA).^233^ PUNISHER was found to be significantly upregulated in the plasma of patients with stable CAD compared to controls while studies on human coronary artery cells showed that pro-atherogenic factors such as oxLDL or TNF-*α* caused the upregulation of PUNISHER levels.^188^ Endothelial activation, monocyte attachment, and transmigration are hallmarks in the development of CAD.^234^ EV-lncRNAs have been reported to take part in the communication between ECs and immune cells.^235^ Monocyte-derived EV-lncRNA **Coromarker** (or AC100865.1) was shown to be upregulated in EVs isolated from plasma of CAD patients compared to controls. In the validation cohort of 221 stable CAD patients and 187 non-CAD controls, Coromarker exhibited 78.05% sensitivity and 86.49% specificity in discriminating CAD.^189^ **H19**, which is under the regulation of insulin growth factors, promotes senescence in cardiomyocytes, and is normally expressed during embryologic development and downregulated later in adult life^198–200^; however, in patients with CAD, re-expression of lncRNA **H19** is observed as indicated by its high levels both in plasma and peripheral blood mononuclear cells (PBMC).^201,202^ Thus, PBMCs could react to certain pathologic stimuli by releasing H19 to the periphery. In another CAD cohort of 137 patients, circulating lncRNA **Upperhand** (also known as HAND2-AS1), known to regulate Hand2 expression during heart development, was significantly upregulated compared with 115 non-CAD controls and exhibited high sensitivity and specificity in diagnosing CAD (0.737 and 0.652 respectively).^203,204^ It should be noted that even though H19 and Upperhand levels were detected in human plasma and whole blood, respectively, none of these studies’ protocols included separate EV isolation steps and thus, whether these lncRNAs are indeed harbored within EVs remained unconfirmed. Furthermore, it has been demonstrated that PUNISHER, H19, and Upperhand are also involved in other pathophysiological processes such as human cancer. These confounding associations which could diminish their diagnostic and prognostic biomarker potential have remained unexplored in the current CVD studies.

### (ii) Acute myocardial infarction (AMI)

ASCVD is driven by lipid accumulation in the arterial wall, inflammation, and vascular injury that lead to coronary arterial stenosis, plaque formation, and AMI.^236^ EV-lncRNAs are involved in all aspects of this atherothrombotic spectrum.^237^ Currently, lncRNA urothelial carcinoma associated 1 (**UCA1**) is one of the very few lncRNA molecules that have been studied in human plasma-derived EVs and are associated with AMI in patient cohorts. In fact, the extracellular levels of UCA1 were significantly higher in AMI patients compared to healthy volunteers, while the AUC for AMI prediction was 0.82, indicating that human plasma extracellular UCA1 has the potential to be a non-invasive biomarker for the diagnosis of AMI.^217^ However, attention needs to be drawn to the confounding potential of certain cancers, such as colorectal cancer, that can also increase circulating UCA1 levels in the serum of patients.^238^ Function-wise, UCA1 was found to suppress miR-873, which in turn increases X-Linked Inhibitor Of Apoptosis (XIAP) levels and promotes apoptosis via the AMPK phosphorylation pathway.^217^ Zinc finger antisense 1 (**ZFAS1**) along with Cdr1 antisense (**CDR1AS**) were significantly different in whole blood samples of AMI and controls. Interestingly, the logistic regression analysis showed that the combination of reduced ZFAS1 levels and increased CDR1AS improved the predictive value for AMI.^209^ Cellular CDR1AS upregulates two apoptotic genes, poly (ADP-ribose) polymerase (PARP) and Specificity protein 1 (SP1), by sponging miR-7a, in myocardial cells, during MI injury.^213^ Cellular ZFAS1 has a dual mechanism of action on cardiomyocytes — inhibiting the sarcoplasmic reticulum Ca2+ ATPase 2a (SERCA2a) after AMI^210^ and activating the mitochondrial apoptosis pathway.^211^ Although the combination of circulating ZFAS1 and CDR1AS was shown to exhibit a diagnostic and prognostic value for AMI, there are still a lot of obstacles to overcome to be clinically utilized. First, similar to several other lncRNA markers, both ZFAS1 and CDR1AS were found to be upregulated in cancer patients,^239,240^ suggesting that cancer is a confounder and might hamper ZFAS1 and CDR1AS diagnostic specificity. Secondly, two separate independent studies showed that, circulating ZFAS1 levels were associated with HF^212^ and type 2 diabetes (T2D)^241^ limiting its diagnostic utility in AMI, given that T2D is strongly associated with AS and AMI.^242^ It is also worth noting that both ZFAS1 and CDR1AS were detected as circulating in whole blood, and the question of whether can be found in the EV compartment was left unanswered. In another cohort of 58 STEMI patients and 50 UA controls, plasma levels of pro-fibrotic EV-lncRNA myocardial infarction associated transcript (**MIAT**) were shown to be elevated in patients with AMI compared to controls. MIAT was positively associated with cardiac dysfunction enzymes, creatine kinase-MB and cardiac troponin T (cTnT). After ROC analysis was performed, it revealed that MIAT had the same diagnostic value as cTnT.^214^ Mechanistically, MIAT exhibits a pro-apoptotic effect in cardiomyocytes by sponging miR-22-3p and, in turn, upregulating the death-associated protein kinase 2 (DAPK2).^215^ Although MIAT is purported in many studies as a specific biomarker for AMI, in another study MIAT was found to be upregulated in the plasma and EVs of patients with atrial fibrillation (Afib). Notably, none of those patients had a prior history of AMI.^216^ Moreover, another study found that MIAT levels are also higher in patients with multiple sclerosis (MS), a neurodegenerative disorder.^243^ These data suggest that MIAT levels could fluctuate responding not only to ischemic stimuli and cardiac injury, but also to other diseases. Nevertheless, to answer the question of whether MIAT can be used clinically as a biomarker for AMI necessitates larger trials that include both AMI and Afib patients as well as neurologic diseases to be assessed as possible confounding factors. Finally, EV-lncRNA **HOTAIR**, which acts by sponging miR-126-5p, is another case of EV-lncRNA that showed utility as a biomarker in CVD as its levels were found to be markedly elevated in blood samples of patients with CAD and AMI^218,219^. However, numerous other conditions, especially neoplastic disorders,^244^ have also been shown to significantly increase its levels in plasma and, therefore hinder the potential of HOTAIR to be a specific biomarker for CVD. Thus, further larger studies are needed to assess HOTAIR specificity for diagnosing AMI. Notably for all these studies, comparisons need to be made to the current gold standard biomarker for MI diagnosis (high sensitivity cardiac troponin T, cTnT) to determine if any of these EV-lncRNAs can add to the diagnostic or prognostic performance of cTnT. Finally, given that myocardial infarction is an acute condition, timely detection, diagnosis, and treatment are of the essence. However, due to the unpredictable nature of such an event data on changes in EV-lncRNA levels immediately following an MI are limited. Additionally current biomarkers of acute coronary syndrome such as high sensitivity troponin are excellent biomarkers, suggesting that the role of EV-lncRNAs as diagnostic markers may be limited, although their role as prognostic markers of post-MI sequelae need to be established. Further research is needed to determine the dynamics of EV-lncRNAs expression and their association with post-MI outcomes.

### (iii) Heart Failure (HF)

In the aftermath of AMI; fibrosis and myocardial scarring lead to ventricular remodeling, which, in turn, triggers arrhythmia and HF.^245^ EV-lncRNAs have been shown to be key players in cardiac tissue remodeling and HF development and thus can be used for diagnostic and prognostic purposes in both chronic and acute setting.^76^ Recently, novel EV-lncRNAs with mechanisms that converge on the nuclear factor of activated T-cells (NFAT) pathway have been implicated in HF. NFAT is a molecule with pivotal functions in regulating intracellular Ca^2+^ homeostasis in HF and pathological hypertrophy.^246,247^ The expression levels of lncRNA cardiac hypertrophy-associated transcript (**CHAST**), which is a transcriptional target of NFAT, were increased acutely and significantly in patients with AMI, and correlated with cardiac systolic function. CHAST was found to be an independent predictor of cardiac contractile function and thus could serve as a clinically useful biomarker for cardiac remodeling.^220^ Furthermore, CHAST has been implicated in cardiac hypertrophy pathways where it was found to suppress Pleckstrin homology domain-containing protein family M member 1, inhibit cardiomyocyte autophagy, and thus promote hypertrophy.^221^ Although more studies are needed, to date, CHAST has not been associated with other diseases besides cardiac hypertrophy and HF, signifying its promising potential for clinical utility in the future. Other EV-lncRNAs implicated in the NFAT pathway, namely., non-coding repressor of NFAT (**NRON**) along with myosin heavy chain related to RNA transcription (**MHRT**) were found to be significantly upregulated in plasma of patients with HF compared to non-HF controls. In fact, ROC analysis showed the area under the curve for NRON and MHRT to be 0.865 and 0.702 respectively. Regression analyses identified NRON and MHRT as independent predictors of HF, demonstrating that both can serve as HF diagnostic biomarkers.^222^ However, it should be noted that NRON was also found to be upregulated in the plasma of patients with MS.^243^ Mechanistically speaking, animal studies have demonstrated cellular lncRNA NRON to be involved in cardiac hypertrophy and myocardial ischemia via hypoxia inducible factor 1a (HIF-1a) modulation.^223,248^ On the other hand, lncRNA MHRT has been found to exert somewhat ambiguous effects on myocardial tissue, either alleviating cardiac hypertrophy, in some studies^224,225^ or promoting cardiac fibrosis after MI through miR-3185.^226^ Finally, another circulating lncRNA **long intergenic noncoding RNA predicting cardiac remodeling** (**LIPCAR**,also known as uc022bqs.1) was shown, in two separate studies, to be a predictor for adverse outcomes both in patients with AMI and chronic HF, since its levels were associated with LV cardiac remodeling and a higher risk for cardiovascular death.^227,228^ In par with those results, several studies found circulating LIPCAR levels to be significantly elevated in patients with HF and showed a positive correlation with NYHA class, NT-proBNP, and high-sensitivity cTnT while negatively associated with left ventricular ejection fraction (LVEF). Furthermore, after ROC analysis, LIPCAR was demonstrated to have a better diagnostic value (AUC: 0.985) of HF following AMI during hospitalization than NT-proBNP (AUC: 0.714).^229,230^ Although its place in AMI or HF diagnosis remains questionable, LIPCAR may have excellent prognostic and predictive value for AMI and HF patients in a clinical setting, and thus, further clinical studies are warranted.

## 5. Neurodegenerative diseases

Advanced age inevitably leads to the loss of neuronal structure and function, which is the hallmark of neurodegenerative diseases (NDD). NDD (Alzheimer’s disease; AD, Parkinson’s disease; PD and motor neuron diseases including amyotrophic lateral sclerosis; ALS) collectively affected almost 6.0 million individuals in the U.S. between 2016–2017. These diseases were responsible for almost 273,000 deaths and 3.0 million disability-adjusted life years in 2016.^249–251^ Clinical biomarkers for early detection and diagnosis of NDDs are currently lacking. Therefore, it is paramount that reliable markers for diagnosis, prognosis, and monitoring disease activity be discovered.

All cells that comprise the central nervous system, including neurons, astroglia, microglia, and oligodendrocytes, release EVs.^252–255^ Studies investigating the potential of EV ncRNAs in NDD diagnosis have largely focused on the miRNA cargo of EVs and have shown promising potential.^256^ On the other hand, lncRNAs have been shown to play major roles in the physiological development of the human brain, exhibit brain region- and cell-type-specificity, suggesting that their perturbations might be central to NDD pathophysiology.^257^ Thus, these characteristics deem EV-lncRNAs as potential circulating diagnostic biomarkers. Hereafter, we review the potential applications of circulating lncRNAs as biomarkers and probes of NDDs in clinical practice.

### (i) Alzheimer’s Disease

Alzheimer’s disease (AD) is the most common form of dementia worldwide and accounts for 60%–80% of all dementia cases.^258^ AD is characterized by progressive cognitive decline and memory deficits. Anatomical manifestations include brain volume loss mainly due to hippocampal degeneration, whereas pathological examination of brain tissue reveals intracellular neurofibrillary tangles rich in hyperphosphorylated Tau and extracellular amyloid-*β* (A*β*) deposition leading to senile plaque formation. A*β* deposition is associated with neurodegeneration, inflammation, and neuronal cell death in AD.^259^ *β*-secretase-1 (BACE1) cleaves amyloid precursor protein (APP) to generate the amyloid-*β* 1–42 and amyloid-*β* 1–40, one of the inciting events in AD pathophysiology. The **antisense transcript of BACE1** (lncRNA **BACE1-AS**) was shown to upregulate BACE1 and increase A*β* expression in patients with AD through a feed-forward regulatory mechanism. BACE1-AS was also elevated in the brain tissue of patients with AD and APP transgenic mice.^260^ Circulating BACE1-AS, which acts by sponging miR-214-3p, was elevated in a small cohort of 35 AD patients.^261^ In another study with 72 AD participants, circulating BACE1-AS levels in sEVs were significantly higher compared to 62 controls. ROC curve analysis demonstrated that the AUC for AD prediction was 0.761, the sensitivity was 87.5%, and the specificity was 61.3%. In the same study, when BACE1-AS expression data was combined with the volume and thickness of the right entorhinal cortex measured by MRI, the specificity and sensitivity were raised to 96.15 and 90.91%, respectively, suggesting that BACE1-AS levels combined with imaging data may serve as a potential diagnostic biomarker for AD.^262^ Towards the purpose of biomarker discovery in neurological diseases, EVs have successfully been isolated from cerebrospinal fluid (CSF).^263^ In a study that enrolled 120 AD patients, 120 PD patients and 120 healthy controls lncRNA **MALAT1** was measured along with miR-125b, FOXQ1, PTGS2, and CDK5 in both CSF and plasma by RT-qPCR. MALAT1 was found to be downregulated both in plasma and CSF of AD patients compared to PD patients and controls. In fact, MALAT1 was able to differentiate AD patients from PD patients (AUC: 0.892) and controls (AUC: 0.830). In AD patients, correlations with mini-mental state exam (MMSE) score, and proteins A*β*42, t-tau and p-tau showed that MALAT1 and FOXQ1, in plasma and CSF, correlated with decreased disease severity, while miR-125b, PTGS2 and CDK5 correlated with increased disease severity.^264^ On the other hand, serious specificity concerns arise when considering the influence of cancer^265^ and other prevalent conditions on MALAT1 levels. In that regard, large cohorts are needed to investigate the associations between MALAT1 and confounding factors such as sex, age, and systemic diseases.^266^

### (ii) Parkinson’s disease

PD is the second most common neurodegenerative disorder characterized by progressive motor dysfunction in older individuals, often accompanied by cognitive abnormalities^267^ with around 2%–3% prevalence in individuals aged more than 65.^268^ The most common feature of PD is the degeneration of dopaminergic neurons in the mid-brain area called substantia nigra (SN), which consequently causes striatal dopaminergic deficiency.^268^ This is caused by the formation of Lewy bodies in the cytoplasm of these dopaminergic neurons attributed to the aggregation of *α*-synuclein leading to clinical symptoms usually associated with motor functions such as bradykinesia, resting tremors, rigidity, and motor failure accompanied by cognitive decline and even dementia.^269,270^ However, since many of these symptoms are found in other neurodegenerative diseases, they are often misdiagnosed because the exact mechanism of PD is still poorly understood. Therefore, identifying appropriate diagnostic biomarkers for this disease is an unmet need, and EV-lncRNAs could offer a promising biomarker role. One of the first studies to explore the EV-lncRNA role was done by Wang et al. wherein they compared the transcriptome of plasma-derived EVs obtained through ultracentrifugation in age and sex-matched 7 PD patients along with 7 healthy controls using RNA-sequencing.^271^ On applying log2 fold-change ± 1.5 and p ≤.05, they identified 24 downregulated and 15 upregulated lncRNAs in the PD group. They further chose to explore **lnc-Makorin Ring Finger Protein 2-42:1 (lnc-MKRN2-42:1)**, downregulated in PD patients, by bioinformatically identifying its mRNA targets, such as EIF4E, MKNK1, BTD, and TMEM78. This was in turn validated in 32 PD patients along with 12 healthy controls using qPCR, with some of them yielding promising results. Although this study is not an extensive characterization for identifying EV-lncRNA biomarkers for PD, it offers a glimpse of the approach. Certainly, further exploration, along with validation in larger cohorts, could help ascertain useful biomarkers. More interestingly, Zou et al. pursued a more focused approach toward biomarker identification.^272^ Based on PD pathology, *α*-synuclein aberrant aggregation plays a pivotal role in the development of this disease. Certain studies have observed the release of *α*-synuclein in EVs in mouse models of PD^273^ and further, EVs derived from brain tissue of dementia seem to be able to contribute to the formation of Lewy bodies demonstrated by *in vitro* and *in vivo* models.^274^ To further probe the mechanism of such EVs, Zou et al. performed a microarray analysis in neuronal-derived EVs with L1 cell adhesion molecules (L1CAM) isolated from blood using antibody-based capture, that was also positive for *α*-synuclein. Based on this, **Long Intergenic- POU class 3 homeobox 3** (**Linc-POU3F3)**, known for its role in the autophagic lysosomal pathway, was selected for further analysis.^272^ Upon comparing the levels of plasma-EV derived Linc-POU3F3 in 93 PD patients and 85 controls, this lncRNA was upregulated in PD patients, while *α*-synuclein was also elevated in these EVs. *β*-Glucocerebrosidase (GCase) has been reported to be associated with *α*-synuclein proteostasis and correspondingly, the GCase activity was shown to be significantly downregulated in PD patient plasma. Each of these lncRNAs exhibited good diagnostic capabilities for PD, with Linc-POU3F3 having an AUC of 0.763, for *α*-synuclein was 0.616, GCase activity 0.684, and all these three combined had an AUC of 0.824 with a sensitivity of 71%, and a specificity of 83% at a cutoff of 0.66. Additionally, Linc-POU3F3 levels correlated with disease severity, however, its biomarker role has to be further researched to be translated at a clinical level. In another approach, Elkouris et al. probed the role of lncRNA in PD patient samples by examining bioinformatically genes proximal to lncRNAs (less than 0.5 kilobases) and closely involved with PD, such as GBA1, SNCA (PARK4), UCH-L1 (PARK5), PINK1 (PARK6), DJ-1 (PARK7), LRRK2 (PARK8), and MAPT.^275^ When they examined the expression of these lncRNAs, namely., SNCA-AS1, AK127687, UCHL1-AS1, PINK1-AS1, AX747125, GBAP1, and MAPT-AS1 in substantia nigra of healthy and PD patients, all of them except GBAP1 were significantly downregulated in PD. These lncRNAs were then assessed in PBMC (Peripheral Blood Mononuclear Cells) and in the EVs derived from CSF using ultracentrifugation. Four out of the six lncRNAs, ***α*-Synuclein Antisense transcript 1 (SNCA-AS1)**, **Microtubule Associated Protein Tau Antisense transcript 1** (**MAPT-AS1)**, **AK127687**, and **AX747125,** were detected in the CSF EVs. However, a more in-depth analysis is required to gauge their biomarker role. The exploration of EV-lncRNAs in PD is still in nascent stages, and further studies are required to identify a suitable clinical biomarker for this disease.

### (iii) Amyotrophic Lateral Sclerosis

Amyotrophic Lateral Sclerosis (ALS) is an adult-onset, progressive neurodegenerative disorder characterized by loss of motor neurons in the cerebral cortex with fatal outcomes. Since the pathogenesis of ALS is still unclear, any insight and clinical biomarkers are urgently required. Although several studies have explored the role of lncRNAs in ALS, few studies have investigated the role of EV-derived lncRNAs. In a recent study by Sproviero et al. transcriptomic analysis was performed on plasma-derived small (sEVs) and large EVs (lEVs) isolated using ultracentrifugation from patients with neurodegenerative diseases such as AD (*n* = 6), PD (*n* = 9), Sporadic ALS (*n* = 6), frontotemporal dementia/FTD ( *n* = 9)^276^ as well as control patients (*n* = 6). Interestingly, the EVs from ALS patients were larger in size than those from other NDD’s. Furthermore, the number of EVs in ALS and FTD patients was higher than in controls. Moreover, this study reported that sEVs had a significantly higher number of differentially expressed long RNAs in ALS than lEVs. In addition, this study failed to identify DEGs in AD and PD; however, several targets were found to be differentially expressed between ALS and FTD, including 9 lncRNAs that were upregulated and 4 that were downregulated. While a major limitation of this study is that it does not validate its results through experimental methods such as qPCR. It is evident that further research is needed to identify appropriate EV-lncRNA biomarkers for ALS.

**Fig. 4.**
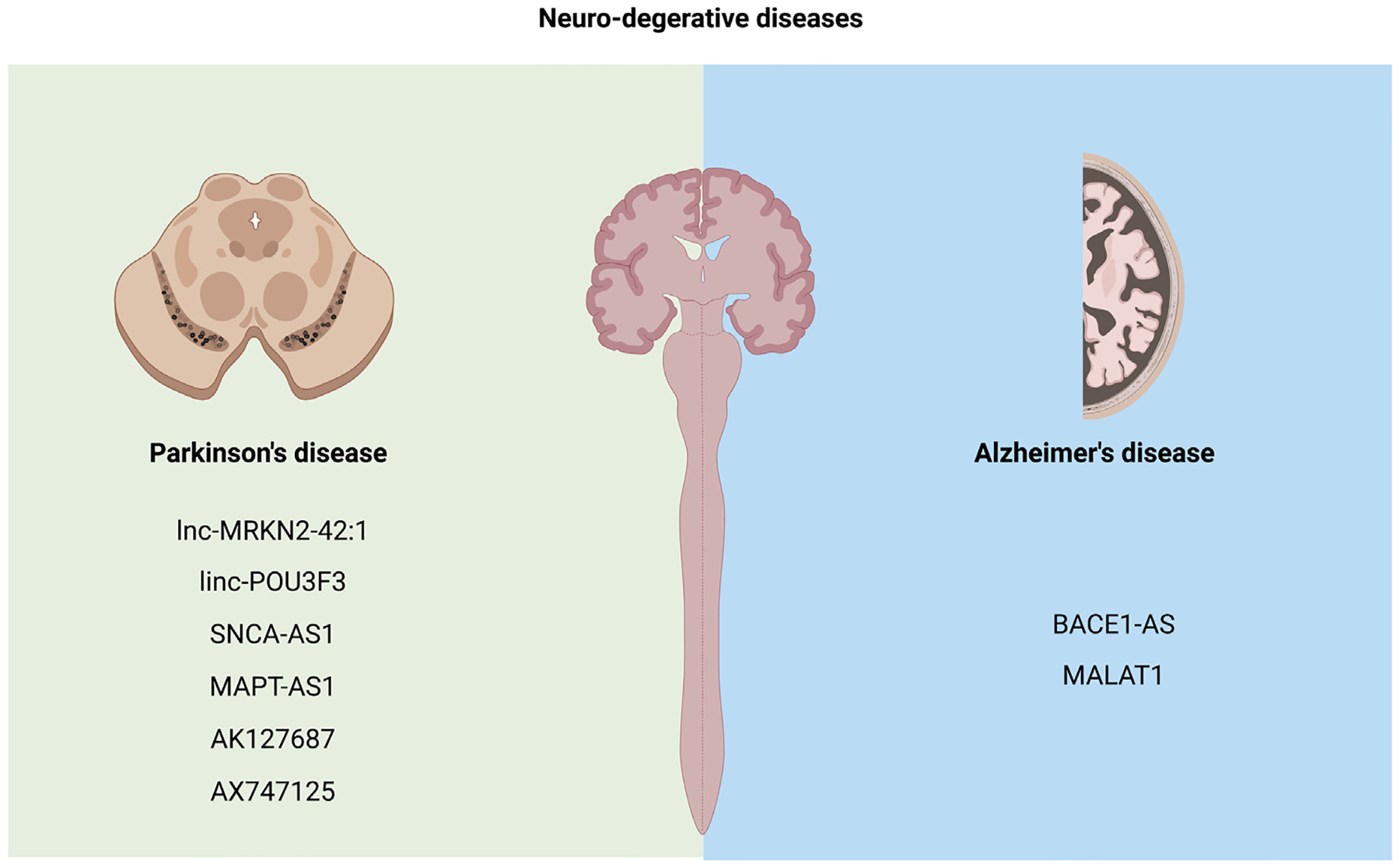
EV-lncRNAs in neuro-degenerative diseases.

Compared to cancer and cardiovascular diseases, there are fewer EV-lncRNAs that have been identified and studied in the context of NDDs (Table 3, Fig. 4). Certainly, extensive research should be conducted before these EV-lncRNAs can be utilized as biomarkers for NDDs in clinical practice.

## 6. Challenges in EV-lncRNA research

Given that both research on EVs and lncRNAs are ever-expanding with room for greater clarity concerning their classification, function, and myriad roles in normal physiology and pathogenesis, extracellular RNA research is perhaps doubly challenged by the limitations of each of these fields. Firstly, the EV isolation method used in each case has significant implications on the outcome of the downstream analysis.^277^ Hence, the lack of standardized isolation protocols poses a challenge in comparing findings made between different research groups. The commercially available isolation kits diminish but do not eliminate the reproducibility problem. Secondly, identifying specific markers for EVs is still challenging, despite extensive research done in this direction. Newer methodologies that not only characterize but also visualize intact EVs more clearly, such as super-resolution microscopy and better classification systems, are still the need of the hour. Third, the annotation of lncRNAs is currently insufficient. While many large-scale efforts to refine current annotations are on the way, annotating the profiled lncRNAs is based on several different databases which might have implications on biomarker validation efforts between different groups. Fourth, while RNA-sequencing (RNA-seq) has replaced microarray technology due to a wider range of RNA quantification and higher throughput capabilities, there is no single RNA-seq method that allows simultaneous profiling of all RNA species. Depending on the RNA-seq technology used, different RNA biotypes can be profiled, however the sequencing method needs to be aligned with the experimental needs, aims and design. Moreover, profiling lncRNA expression in biofluids presents additional challenges. In human plasma, extracellular lncRNAs are usually found in fragments, which in turn necessitates a thorough library preparation procedure. It is still unclear whether the long RNA species is present as fragments or full-length in EVs. Especially in the case of cellular stress, lncRNAs could be packaged in EVs to prevent any toxic build-up to improve cell-viability and consequently be fragmented. Interestingly, in some cases, cells subjected to stress preferentially increase the expression of a certain lncRNA fragment in the EVs, suggesting that the fragmentation itself is not a random process and seems to follow a specific mechanism (unpublished observations). While there are emerging reports of fragments in EVs, it is unclear if the fragmentation is a way of clearance from the cell or if the fragments themselves have a functional role.^278^ In fact, the functionality of lncRNA fragments is only recently explored, and its specific role in EVs is currently unknown. Most reports focus only on the functionality of the lncRNAs packaged in EVs, but have not critically evaluated the nature or the function of the EV-lncRNA itself. Finally, studying the mechanism of long RNA packaging in EVs is a huge challenge given the different types of s-EVs and a possible differential cargo. Therefore, most studies have focused on small RNA packaging in EVs, with very few exploring the mechanism of long RNA packaging in EVs. Given that this is a huge unmet need in the field, hopefully, future studies focus on studying this aspect of EV-lncRNAs.

## 7. Conclusions and future perspectives

While this review offers a glimpse of what lies ahead for EV-lncRNA research in diseases, one can conclude that EV-derived lncRNAs and their fragments have a dynamic expression and are highly sensitive to physiological changes aiding in their promising biomarker role, sometimes even outperforming the conventional protein biomarkers. However, in light of these challenges, we should focus on identifying and understanding more precisely how these EV-lncRNAs function in diseases, how they change in response to disease progression or therapy, and what associations with clinical outcomes in order to have a more targeted approach to theranostics and prognosis. Finally, probing disease-related EV-lncRNAs may provide insight into novel druggable pathways and targets in the cells of origin.

## Figures and Tables

**Table 1:** EV lncRNAs in Cancer.

Cancer type	lncRNA	Expression	Function	EV isolation method	Biofluid	Type of study	Validated in (number)	AOC curve	Reference
Non-small cell lung cancer (NSCLC)	MALAT-1	Upregulated	Oncogene	ExoQuick TC	Serum	qPCR, cell culture validated	Control (30) and NSCLC (77)	0.703	Ref. 112
	MALAT-1	Upregulated	oncogene	ExoQuick precipitation kit	Serum	qPCR, cell culture validated	Control (52) and NSCLC (52)	NA	Ref. 113
	DLX6-AS1	Upregulated	oncogene	ExoQuick precipitation kit	Serum	qPCR	Control (64) and NSCLC (72)	0.806	Ref. 114
	LINC00662	Upregulated	oncogene	ExoQuick precipitation kit	Plasma	qPCR, cell culture validated	Control (50) and NSCLC (50)	NA	Ref. 115
	UFC1	Upregulated	oncogene	NA	Serum	qPCR, cell culture validated, in vivo	Control (40) and NSCLC (54)	0.794	Ref. 116
	SOX2OT	Upregulated	Bone metastasis	Ultracentrifugation	Plasma	qPCR, cell culture validated	NSLC(142) and BoM	NA	Ref. 117
	H19	Upregulated	drug resistance	ExoQuick precipitation kit	serum	qPCR, cell culture validated	Responding(28) and nonresponding 30)	0.799	Ref. 118
	RP11-838N2.4	Upregulated	drug resistance	ExoQuick precipitation kit	serum	qPCR, cell culture validated	Responding(43) and nonresponding (35)	0.754	Ref. 119
	HOTTIP	Upregulated	Prognostic marker	ExoQuick precipitation kit	serum	qPCR	Relapsed (7) and recovered (11) post-surgery and therapy	0.935	Ref. 120
	GAS5	Downregulated	Diagnostic marker	Exosome Isolation Kit			Control (40) and NSCLC (64)	0.857	Ref. 121
	TBILA, AGAP2-AS1	Upregulated	Diagnostic marker	ExoQuick precipitation kit	Serum	qPCR	Control (100) and NSCLC (100)	0.775,0.734	Ref. 122
Lung Squamous Cell Carcinoma (LSCC)	SOX2OT	Upregulated	oncogene	Ultracentrifugation	Plasma	qPCR, cell culture validated	Control (79) and LSCC (72)	0.815	Ref. 123
Colorectal Cancer (CRC)	UCA1	Downregulated	Diagnostic marker	ExoQuick TC	Serum	qPCR	Control (20) and CRC (20)	0.718	Ref. 124
	CRNDE-h	Upregulated	Diagnostic marker	ExoQuick TC	Serum	qPCR	Control (80) and CRC (148)	0.892	Ref. 125
	LINC00659	Upregulated	Oncogene	Ultracentrifugation	CAF Evs	qPCR	NF (8) and CAF (8)		Ref. 126
	ADAMTS9-AS1	Upregulated	Diagnostic marker	Ultracentrifugation		qPCR	Control (130) and CRC (130)	0.855	Ref. 127
	HOTTIP	Upregulated	Diagnostic marker	Thermofisher Exosome Isolation Kit		qPCR	Control (52) and CRC (52)	0.75	Ref. 128
	CCAL	Upregulated	Oncogene		CAF Evs	qPCR	NF (15) and CAF (15)		Ref. 129
	H19	Upregulated	Oncogene	ultracentrifugation	CAF Evs	qPCR	NF (10) and CAF (10)		Ref. 130
	GAS5	Downregulated	Diagnostic and prognostic, tumor supressor	ultracentrifugation	Plasma	qPCR	Control (178) and CRC (153)	0.961	Ref. 131
	XIST	Upregulated	Diagnostic and prognostic	ultracentrifugation	Serum	qPCR	Control (41) and CRC (94)	0.864	Ref. 132
Breast Cancer (BC)	H19	Upregulated	Diagnostic marker	NA	Plasma	qPCR	Control (96) and BC (102)	0.81	Ref. 133
	H19	Upregulated	drug resistance	ExoQuick TC	serum	qPCR	Responding(36) and non responding (46)	0.752	Ref. 134
	XIST	Upregulated	Diagnostic, prognostic, recurrence	Exosome Isolation Kit	serum	qPCR	Control(50) and TNBS(91)	0.888	Ref. 135
	HOTAIR	Upregulated	diagnostic, prognostic, drug-resistance	ExoQuick TC	serum	qPCR	Control(15), BC(15)	0.916	Ref. 136
	DANCR	Upregulated	diagnostic, prognostic, drug-resistance	Ultracentrifugation	serum	qPCR	Control(105), Benign breast disease(70), BC(120)	0.88	Ref. 137
	H19	Upregulated	diagnostic, prognostic, drug-resistance	Exosome Extraction Reagent	serum	qPCR	Control(50), Benign breast disease(50), BC(50)	0.87	Ref. 138
	AGAP2-AS1	Upregulated	drug resistance	ExoQuick TC	serum	qPCR, cell culture validated, in vivo	Her2+ BC-Responding(45) and non responding (45)	0.784	Ref. 139
	MALAT1	Upregulated	oncogene	ultracentrifugation	serum	qPCR, cell culture validated, in vivo	BC (80)	NA	Ref. 140
Triple Negative Breast Cancer (TNBC)	SUMO1P3	Upregulated	prognostic marker	ultracentrifugation	serum	qPCR	Control(30) and BC(90, stage I-30,II-30,III-30)		Ref. 141

**Table 2:** EV lncRNAs in CVD.

lncRNA	Disease	Status	Function	Origin	Study design	Validation	References
SNHG9	CAD, Obesity, PAD	reduced	TRADD gene suppression	plasma, Adipose-derived stem cells (ADSCs) and Human umbilical vein endothelial cells (HUVECs)-derived EVs	Cohort study; Control group normal-weight (*n* = 32), obese-only (*n* = 17); obese with endothelial dysfunction (DB, *n* = 18; CAD, *n* = 23; PAD, *n* = 16; HBP, *n* = 15)		Ref. 187
PUNISHER	CAD	increased	Modulates VEGFA activity	plasma s Evs	Cohort study; Total cohort *n* = 221; lncRNA profiling cohort (CAD; *n* = 3, controls; *n* = 3) Validation cohort (CAD; *n* = 30, controls; *n* = 30)	(CAD; *n* = 30, controls; *n* = 30)	Ref. 188
Coromarker	CAD	increased	Unknown	plasma, monocytes	Cohort study; CAD group *(n* = 221); non-CAD control group (*n* = 187)	CAD group (*n* = 221) non-CAD control group *(n* = 187)	Ref. 189
TONSL-AS1	CAD	reduced	upregulates BCL-2	Plasma	Cohort study; CAD group *(n* = 60); non-CAD control group (*n* = 60)		Ref. 190
MALAT1	CAD	increased	Induction of M2 phenotype in macrophages, p38/AKT signaling pathway activation, NLRP3 modulation, miR-497 suppression	serum, arterial tissue, endothelial cells, HUVEC-derived Evs	Cohort study; CAD group *(n* = 20)		Refs. 191–193
HIF1A-AS1	CAD	increased	Induced by Brahma-related gene 1 (BRG1), promotes apoptosis and inhibits proliferation of VSMCs	plasma s Evs	Cohort study; AS group *(n* = 65); non-AS group *(n* = 68)		Refs. 194–197
H19	CAD	increased	Regulated by IGF-2, suppresses SOCS1 expression by sponging miR-19a, promotes cardiomyocyte senesence	plasma	Cohort study; CAD group (*n* = 300) ; control group *(n =* 180)		Refs. 198–202
Upperhand	CAD	increased	Regulates Hand2 expression	whole blood	Cohort study; Total cohort *n* = 324; Discovery cohort: microarray analysis (CAD; *n* = 6, controls; *n* = 6),	Validation cohort 1; (CAD; *n =* 30, controls; *n* = 30) Validation cohort 2; (CAD; *n* = 137, controls; *n* = 115)	Refs. 203, 204
CHROME	CAD	increased	Regulates d miRNAs miR-27b, miR-33a, miR-33b and miR-128	plasma	Cohort study; CAD group *(n* = 14); control group *(n* = 33)		Ref. 205
NEAT1	STEMI	increased	sponges miR-495-3p/indirectly activates MAPK6 pathway	plasma s Evs	Cohort study; Control group (*n* = 27); UA group (*n* = 24); STEMI group (*n* = 47)		Refs. 206–208
ZFAS1	AMI/HF	reduced	Modulates SERCA2a protein/induces mitochondria-mediated apoptosis via cytosolic Ca2+ overload	whole blood	Cohort study; AMI group (*n* = 103); non-AMI group (*n* = 149); control group (*n* = 95)		Refs. 209–212
CDR1AS	AMI	increased	Sponges miR-7a/upregulates PARP,SP1	whole blood	Cohort study; AMI group *(n* = 103); non-AMI group *(n =* 149); control group (*n* = 95)		Refs. 209, 213
MIAT	AMI	increased	sponges miR-22-3p/upregulates DAPK2	Plasma	Cohort study; STEMI group *(n* = 58); UA control group (*n* = 50)		Refs. 214–216
UCA1	AMI	increased	sponges miR-873/XIAP upregulation/AMPK phosphorylation and increased level of the antiapoptotic protein BCL2	plasma s Evs	Cohort study; AMI group (*n* = 26, ccu patients); control group (*n* = 26)		Ref. 217
HOTAIR	AMI	Decreased	sponges miR-126-5p	Plasma	Cohort study; AMI group *(n* = 50); control group *(n* = 50)		Refs. 218, 219
CHAST	AMI/HF	increased	suppresses Pleckstrin homology domain-containing protein family M member 1	Plasma	Cohort study; AMI group *(n* = 53); control group *(n* = 90)		Refs. 220, 221
NRON	HF	increased	upregulates HIF1a	plasma	Cohort study; HF group *(n* = 72) non-HF control group *(n* = 60)		Refs. 222, 223
MHRT	HF	increased	antagonizes the function of Brg1/suppresses miR-3185/reduces myocardin expression through KLF4 modulation	plasma	Cohort study; HF group *(n* = 72) non-HF control group *(n* = 60)		Refs. 222, 224–226
LIPCAR	STEMI/HF	increased	Unknown	plasma	**HF Cohort study 1**; MI and LV remodeling group *n* = 246 ; **HF Cohort study 2**; STEMI group *(n* = 46); control group *(n* = 40); **HF Cohort study 3**; non-HF post-AMI group (*n* = 68); HF post-AMI group *(n* = 59), 30 control group (*n* = 30) **HF Cohort study 4**; HF group *(n* = 967)	validation cohort 1; chronic HF group *(n* = 344); validation cohort 2; chronic HF group 2 (*n* = 198); STEMI	Refs. 227–230

**Table 3:** EV lncRNAs in NDD.

NDD type	lncRNA	Expression	Source	EV isolation method	Patient Cohort	RNA validation method	AUC	Reference
Alzheimer’s Disease (AD)	BACE1-AS	Upregulated	Plasma	NA	35	qPCR		Ref. 260
	BACE1-AS	Upregulated	Plasma	ExoQuick Precipitation	72	qPCR	0.761	Ref. 261
	MALAT1	Downregulated	PLasma	NA	120 AD, 120 PD, 120 normal	qPCR	0.83	Ref. 263
Parkinson’s disease (PD)	lnc-MKRN2–42:1	Downregulated	Plasma	Ultracentrifugation	Discovery cohort (7 Healthy;7PD), Validation cohort (32 PD; 12 healthy)	qPCR	NA	Ref. 270
	Linc-POU3F3	Upregulated	Plasma	LICAM Pulldown	93 PD patients and 85 controls	qPCR	0.763	Ref. 271
